# Glucagon-Like Peptide-1 (GLP-1)-Based Therapies and Heart Failure Outcomes: A Scoping Review of Contemporary Randomized Controlled Trials

**DOI:** 10.7759/cureus.112239

**Published:** 2026-07-07

**Authors:** Jenika Patel, Deep Patel, Maria Pino

**Affiliations:** 1 Medicine, New York Institute of Technology College of Osteopathic Medicine, Old Westbury, USA; 2 Clinical Specialties, New York Institute of Technology College of Osteopathic Medicine, Old Westbury, USA

**Keywords:** chronic kidney disease, glp-1 receptor agonists, heart failure, hfpef, obesity, scoping review, semaglutide, tirzepatide, type 2 diabetes

## Abstract

Glucagon-like peptide-1 (GLP-1)-based therapies have been observed to produce cardiovascular benefits in cardiometabolic diseases; however, their impact on heart failure-specific outcomes is only becoming known through dedicated randomized controlled trials (RCTs). This scoping review consolidates the most recent randomized data that examine how GLP-1-based therapies affect heart failure-specific symptoms, functional capacity, and the occurrence of clinical events. Literature searches were conducted in PubMed, Google Scholar, and CINAHL for RCTs and prespecified analyses between 2021 and 2026 evaluating GLP-1-based therapies and reporting heart failure-specific outcomes. Five RCT-based studies met inclusion criteria, including randomized trials and prespecified RCT-derived analyses evaluating semaglutide and tirzepatide. Among patients with heart failure with preserved ejection fraction (HFpEF) and obesity, semaglutide significantly improved Kansas City Cardiomyopathy Questionnaire Clinical Summary Score and 6-minute walk distance compared to placebo. Tirzepatide exhibited improvements across symptoms and functional and event-based outcomes, with prespecified trajectory analyses confirming favorable shifts in New York Heart Association functional class, Patient Global Impression of Severity, health-related quality of life, and background heart failure medication use. Among patients with type 2 diabetes mellitus and chronic kidney disease, semaglutide was associated with a significantly reduced rate of cardiovascular death or worsening heart failure events. Although the available evidence comes from a small number of recent randomized and prespecified RCT-based studies, current findings suggest that GLP-1-based therapies are associated with improvements in symptoms and functional capacity and reductions in heart failure events in certain patient populations, especially those with HFpEF and obesity. These beneficial effects support a potential role for the integration of GLP-1-based therapies in population-specific heart failure management.

## Introduction and background

Heart failure is a major cause of morbidity and mortality across the globe. It is a complex condition resulting from structural or functional abnormalities that impair contraction or filling of the heart. In patients with heart failure with preserved ejection fraction (HFpEF), left ventricular (LV) ejection fraction is normal, although contractile dysfunction is often present. Patients with heart failure with reduced ejection fraction (HFrEF) have a prominent LV contraction problem [1]. Heart failure with mildly reduced ejection fraction (HFmrEF) is also recognized as an intermediate ejection fraction category, but it was not analyzed separately in this review because the included studies did not evaluate HFmrEF as a distinct heart failure subgroup. Besides female sex and increasing age, HFpEF is associated with obesity, type 2 diabetes mellitus (T2DM), arterial hypertension, anemia, chronic obstructive pulmonary disease, and chronic kidney disease (CKD) [2]. The prognosis of heart failure is poor, with a five-year mortality of about 75% [3]. Pharmacological therapy, cardiac rehabilitation, and device therapy have improved outcomes in HFrEF, but similar benefits have not been shown to be as consistent in HFpEF [1]. This may partly be due to the complex nature of HFpEF, which is often influenced by obesity, diabetes, hypertension, CKD, inflammation, and vascular stiffness. Pharmacologic therapies such as mineralocorticoid receptor antagonists and sodium-glucose cotransporter 2 inhibitors improve outcomes for HFpEF, but residual risk still persists [4]. Glucagon-like peptide-1 (GLP-1)-based therapies include GLP-1 receptor agonists (GLP-1RAs) and dual glucose-dependent insulinotropic polypeptide (GIP)/GLP-1 receptor co-agonists, which decrease glycated hemoglobin and reduce cardiovascular risk. GLP-1RAs primarily act through GLP-1 receptor signaling, whereas dual GIP/GLP-1RAs, such as tirzepatide, target both GIP and GLP-1 pathways. These therapies were first widely used for T2DM in patients with obesity and atherosclerotic cardiovascular disease and have emerged as a potential alternative treatment for heart failure [5].

Recent reviews indicate that GLP-1-based therapies reduced major adverse cardiovascular events, but their effects on heart failure endpoints were unclear. Reductions in cardiovascular endpoints with GLP-1RAs are largely driven by atherosclerotic risk decline and have neutral or modest effects on heart failure hospitalization. GLP-1RAs may be inconclusive or even harmful in HFrEF. There was no improvement in heart failure rehospitalization in subjects with a history of heart failure [6,7]. In contrast, systematic reviews and meta-analyses of randomized controlled trials (RCTs) showed that GLP-1RAs improved left ventricular diastolic function and heart failure hospitalization in those without a history of heart failure [6]. GLP-1RAs significantly reduced the composite outcome of cardiovascular death and worsening heart failure events as well as heart failure events alone in subjects with HFpEF [4]. These findings are from a limited number of trials with variable outcomes; therefore, it remains ambiguous whether GLP-1RAs have strongly demonstrated benefits for reduction in heart failure endpoints [4,6,7]. GLP-1-based therapies may affect heart failure outcomes through weight loss, improved glycemic control, lower inflammation, and reduced adiposity-related cardiac stress. These effects may be important in HFpEF, where obesity, diabetes, and CKD often contribute to symptoms and disease progression. The 2021-2026 timeframe was chosen because the key randomized evidence on GLP-1-based therapies and heart failure-specific outcomes has largely emerged in recent years, especially in HFpEF and cardiometabolic heart failure populations. This scoping review aims to map current evidence, identify gaps in knowledge, and summarize the findings of contemporary randomized and prespecified RCT-derived studies evaluating GLP-1-based therapies and heart failure-related outcomes. Unlike large reviews that combine different types of heart failure populations, non-heart failure-specific outcomes, or secondary analyses that are overlapping, this scoping review is devoted only to contemporary randomized and prespecified RCT-derived studies publishing heart failure-specific results, and separately discusses symptom-based, functional, and event-based outcomes. The arrival of STEP-HFpEF, STEP-HFpEF DM, SUMMIT, and prespecified heart failure analyses has significantly altered the GLP-1 and heart failure evidence base rapidly; hence, an updated and targeted synthesis is pertinent.

## Review

Methods

Literature searches were conducted on PubMed, Google Scholar, and CINAHL databases. All databases were last searched on May 17, 2026. Screening was completed in two stages: an initial review of titles and abstracts, followed by a comprehensive evaluation of the full-text articles. Rayyan was used to manage the screening and study selection process. Two reviewers independently screened titles and abstracts while adhering to predefined inclusion and exclusion criteria. Articles considered potentially relevant underwent full-text analysis to confirm their inclusion in our study. Any disagreements about study eligibility were discussed together until agreement was reached.

A standardized method was employed to chart the data from the included studies. Data charting was performed independently by two reviewers, and any disputes were settled through discussion and consensus. The factors that were charted included study design, subject information, heart failure population, interventions, comparators, outcome measures, assessment time points, heart failure-specific symptoms, and functional and clinical event outcomes.

Because contemporary randomized evidence evaluating GLP-1-based therapies and heart failure-specific outcomes has emerged primarily in recent years, we limited the search to studies published between 2021 and 2026. This helped keep the review focused on the most current and clinically relevant evidence. In PubMed, a hybrid search strategy incorporating both medical subject headings (MeSH) terms and keyword searching was used. Search concepts included GLP-1-based therapies, semaglutide, tirzepatide, and heart failure. The following search strategy was used in PubMed: ("Glucagon-Like Peptide 1 Receptor Agonists"[Mesh] OR semaglutide[tiab] OR tirzepatide[tiab] OR "GLP-1 receptor agonist"[tiab] OR "GLP-1 receptor agonists"[tiab] OR incretin[tiab] OR incretins[tiab]) AND ("Heart Failure"[Mesh] OR "heart failure"[tiab] OR HFpEF[tiab] OR HFrEF[tiab] OR "heart failure with preserved ejection fraction"[tiab] OR "heart failure with reduced ejection fraction"[tiab]).

Google Scholar was additionally searched to identify potentially relevant studies and recently published articles not yet indexed in PubMed. The following search strategy was used in Google Scholar:("GLP-1" OR "GLP-1 receptor agonist" OR semaglutide OR tirzepatide) ("heart failure" OR HFpEF) (randomized OR placebo OR trial OR "prespecified analysis") (obesity OR diabetes OR CKD OR "chronic kidney disease") -review -meta-analysis -guideline -editorial. The following search strategy was used in CINAHL: ((MH "Glucagon-Like Peptide 1 Receptor Agonists+") OR TI (semaglutide OR tirzepatide OR "GLP-1 receptor agonist" OR "GLP-1 receptor agonists" OR incretin OR incretins) OR AB (semaglutide OR tirzepatide OR "GLP-1 receptor agonist" OR "GLP-1 receptor agonists" OR incretin OR incretins)) AND ((MH "Heart Failure+") OR TI ("heart failure" OR HFpEF OR HFrEF) OR AB ("heart failure" OR HFpEF OR HFrEF)) AND (TI (randomized OR placebo OR trial OR "prespecified analysis") OR AB (randomized OR placebo OR trial OR "prespecified analysis")) NOT (TI (review OR meta-analysis OR protocol) OR AB (review OR meta-analysis OR protocol)).

Publication years ranging from 2021 to 2026 were selected across all three databases for analysis. In PubMed, an RCT filter was applied to further refine the search. However, comparable RCT filters were not used in Google Scholar or CINAHL. As a result, these two databases were restricted to the specified publication years, with subsequent manual screening conducted to determine study design alignment with eligibility criteria. The application of database filters and search parameters preceded manual screening efforts, with these processes documented and represented in the Preferred Reporting Items for Systematic reviews and Meta-Analyses (PRISMA) diagram.

Inclusion criteria included English-language full-text RCTs and prespecified analyses derived from RCTs published from 2021 to 2026 that evaluated GLP-1-based therapies and reported heart failure-specific outcomes. We included prespecified analyses from randomized trials when they reported heart failure-specific outcomes, even if the original trial had a different primary endpoint. For this reason, the FLOW heart failure outcomes analysis met inclusion criteria, while non-prespecified secondary or subgroup analyses were excluded. Ongoing trials were not included in the evidence synthesis because this review was limited to published full-text randomized trials and prespecified RCT-derived analyses available through the final search date. Exclusion criteria included duplicates, reviews, non-randomized designs, non-prespecified secondary analyses or subgroup studies, mechanistic or biomarker-only studies, studies not addressing the outcomes of interest, studies using pediatric populations, studies focused on perioperative, acute, or non-heart failure populations, and studies without GLP-1-based therapy as the randomized intervention. A formal risk-of-bias assessment was not performed because this was designed as a scoping review rather than a systematic review or meta-analysis. This scoping review was conducted in accordance with PRISMA-ScR reporting recommendations [8]. A review protocol was prepared before the review was conducted, but this scoping review was not formally registered.

Results

Literature Search

**Figure 1:**
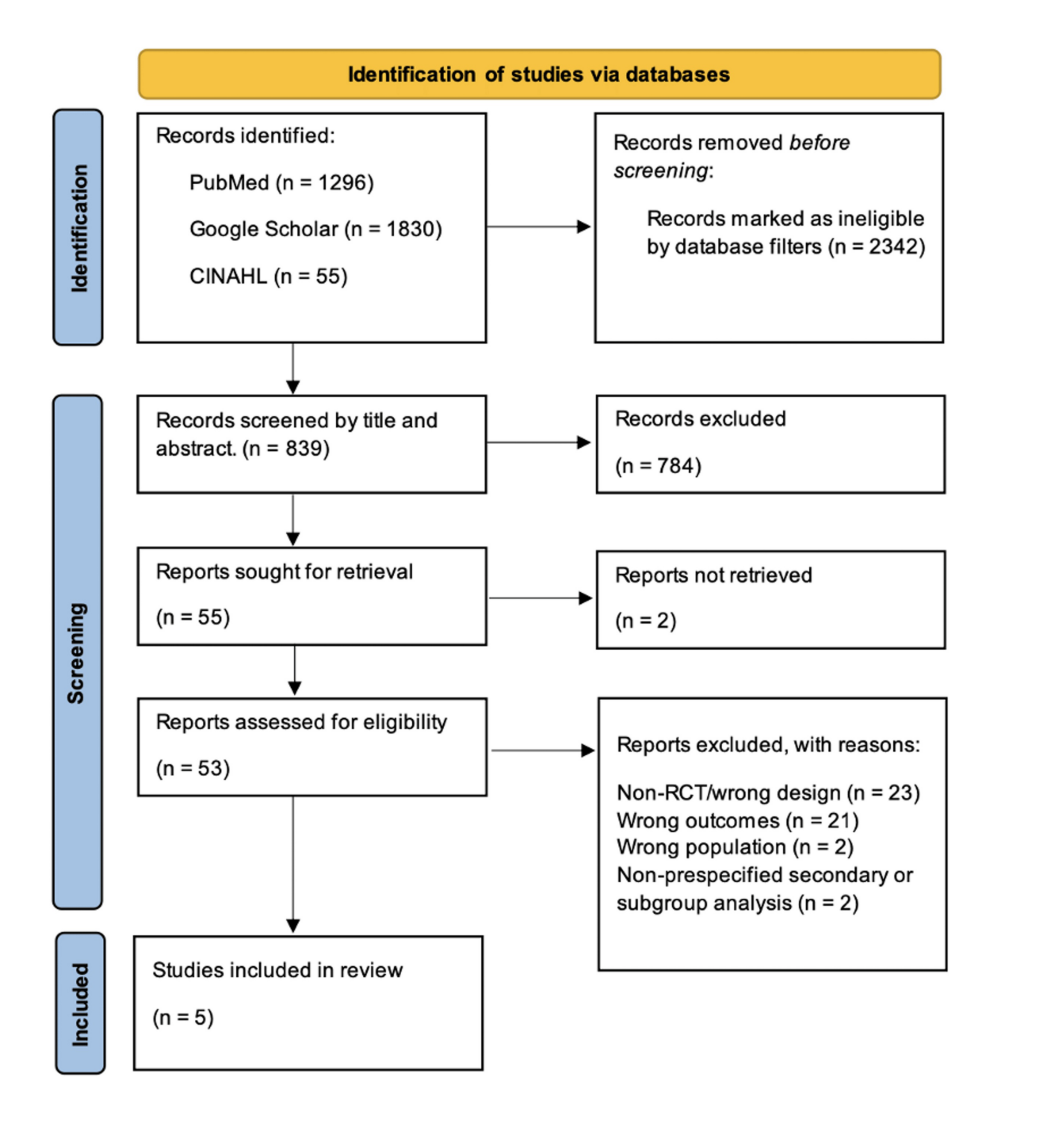
PRISMA flow diagram detailing the screening, eligibility assessment, and selection of studies included in the review. PRISMA, Preferred Reporting Items for Systematic reviews and Meta-Analyses; RCT, randomized controlled trial.

A total of 3,181 records were identified: 1296 from PubMed, 1830 from Google Scholar, and 55 from CINAHL. Following screening and exclusion of articles, five RCT-based studies underwent review (Figure 1).

Information on the five included studies is as follows: The STEP-HFpEF trial by Kosiborod et al. explored the use of semaglutide in patients with HFpEF and obesity [9]. The successive trial by the same team of researchers, STEP-HFpEF DM, studied the impact of semaglutide in a similar HFpEF population where T2DM was added as a comorbidity [10]. Outcomes from both articles were summarized using the treatment policy estimand [9,10]. The SUMMIT trial, reported by Packer et al., aimed to understand the impact of tirzepatide in patients with HFpEF and obesity [11]. A prespecified analysis of the SUMMIT trial by Zile et al. deepened the characterization of the clinical trajectory of the patients enrolled in the parent randomized study [12]. Lastly, the FLOW heart failure study by Pratley et al. reported heart failure outcomes from a randomized trial of semaglutide in patients with T2DM and CKD [13]. Although these studies evaluated related heart failure outcomes, they included different patient populations. Therefore, the findings should be interpreted in the context of the populations studied, especially HFpEF with obesity, HFpEF with obesity and T2DM, and T2DM with CKD.

Measurement Tools

Kansas City Cardiomyopathy Questionnaire Clinical Summary Score (KCCQ-CSS): Scores range from 0 to 100, with higher scores indicating fewer symptoms and physical limitations alongside better quality of life [9-11].

6-minute walk distance (6-MWD): It quantifies the total meters walked in six minutes during a standardized walk test, with greater distances indicating better functional capacity [14].

EuroQol 5-Dimension 5-Level (EQ-5D-5L) health state index: Generic measure of health-related quality of life comprising five dimensions (mobility, self-care, usual activities, pain/discomfort, and anxiety/depression), each assessed at five levels of severity, defining 3125 possible health states that are summarized as a single index value [15].

Patient Global Impression of Severity (PGIS): Single-item, patient-reported measure in which individuals rate the overall severity of their condition using an ordinal scale, commonly ranging from 0 (no symptoms) to 5 (very severe) [16].

New York Heart Association (NYHA) functional class: This categorizes heart failure severity into four classes (I-IV) based on the degree of limitation during physical activity, with Class I indicating no limitation and Class IV indicating symptoms at rest [17].

Outcomes

In total, five RCT-based studies assessing GLP-1-based therapies and heart failure-related outcomes were included (Table 1). Within these trials, heart failure outcomes were evaluated using patient-reported symptom scores, functional capacity, and clinical heart failure events. The studies involved patients with HFpEF and obesity, HFpEF with obesity and T2DM, and patients with T2DM and CKD in which heart failure outcomes were prespecified. The included studies suggest that GLP-1-based therapies may affect heart failure across symptoms, function, and clinical events.

**Table 1:** Key characteristics and outcomes of included RCTs and prespecified RCT-derived analyses Note: Effect estimates are reported as presented in the original studies and include different measures, such as hazard ratios, win ratios, and mean differences. These values reflect different statistical approaches and should not be interpreted as directly comparable effect sizes across studies. 6-MWD, 6-minute walk distance; BMI, body mass index; CKD, chronic kidney disease; CV, cardiovascular; DM, diabetes mellitus; EQ-5D-5L, EuroQol 5-Dimension 5-Level; HF, heart failure; HFpEF, heart failure with preserved ejection fraction; HR, hazard ratio; IV, intravenous; KCCQ-CSS, Kansas City Cardiomyopathy Questionnaire Clinical Summary Score; NYHA, New York Heart Association; PGIS, Patient Global Impression of Severity; RCT, randomized controlled trial; T2DM, type 2 diabetes mellitus.

Reference	Study	Study type	Purpose	Methods	Measures	Outcomes
Kosiborod et al., 2023 [9]	STEP-HFpEF	RCT	To assess how semaglutide affects physical limitations, health-related quality of life, and symptoms in patients with HFpEF and obesity	529 patients who had HFpEF and a BMI of 30 kg/m^2^ or higher were randomly assigned to receive once-weekly semaglutide (2.4 mg) or placebo for 52 weeks.	Primary outcome: change in KCCQ-CSS from baseline to 52 weeks. Secondary outcomes: change in 6-MWD from baseline to 52 weeks; hierarchical composite endpoint included all-cause death (baseline-week 57); HF events (number and timing); KCCQ-CSS change thresholds (≥15, ≥10, and ≥5 points at week 52); 6-MWD improvement ≥30 m at week 52	Primary outcome: semaglutide significantly improved KCCQ-CSS versus placebo at 52 weeks (Δ 7.8 points; p<0.001). Secondary outcomes: semaglutide significantly improved 6-MWD versus placebo at 52 weeks (Δ 20.3 m; p<0.001); semaglutide outperformed placebo on the hierarchical composite endpoint (win ratio: 1.72; p<0.001), with wins favoring semaglutide across all key components.
Kosiborod et al., 2024 [10]	STEP-HFpEF DM	RCT	To evaluate the effect of semaglutide on symptoms of HF and functional status of patients with HFpEF, obesity, and T2DM	616 patients who had HFpEF, a BMI of 30 kg/m^2 ^or more, and T2DM were randomly assigned to receive once-weekly semaglutide (2.4 mg) or placebo for 52 weeks.	Primary outcome: change in KCCQ-CSS from baseline to 52 weeks. Secondary outcomes: change in 6-MWD from baseline to 52 weeks; hierarchical composite endpoint included all-cause death (baseline-week 57); HF events (number and timing); KCCQ-CSS change thresholds (≥15, ≥10, and ≥5 points at week 52); 6-MWD improvement ≥30 m at week 52	Primary outcome: semaglutide significantly improved KCCQ-CSS versus placebo at 52 weeks (Δ 7.3 points, p<0.001). Secondary outcomes: semaglutide significantly improved 6-MWD versus placebo at 52 weeks (Δ 14.3 m, p<0.05); semaglutide outperformed placebo on the hierarchical composite endpoint (win ratio: 1.58; p<0.001), favoring semaglutide for most key components.
Packer et al., 2025 [11]	SUMMIT	RCT	To discover how tirzepatide impacts worsening HF events and clinical outcomes in patients with HFpEF and obesity	731 patients with HFpEF and a BMI of at least 30 kg/m^2 ^were randomly assigned to receive tirzepatide (up to 15 mg subcutaneously once per week) or placebo for at least 52 weeks.	Primary outcomes: composite of adjudicated CV death or worsening HF, analyzed as time-to-first event; change in KCCQ-CSS from baseline to 52 weeks. Secondary outcome: change in 6-MWD from baseline to 52 weeks	Primary outcomes: tirzepatide significantly reduced the risk of CV death or worsening HF versus placebo (HR: 0.62; p<0.05); tirzepatide significantly improved KCCQ-CSS versus placebo at 52 weeks (Δ 6.9 points, p<0.001). Secondary outcome: tirzepatide significantly improved 6-MWD versus placebo at 52 weeks (Δ 18.3 m, p<0.001).
Zile et al., 2025 [12]	SUMMIT (clinical trajectory analysis)	Prespecified RCT-derived analysis	To characterize longitudinal changes in functional status, symptoms, and clinical trajectory in patients with HFpEF and obesity treated with tirzepatide	This prespecified analysis used the randomized population from the SUMMIT trial, which enrolled 731 patients with HFpEF and a BMI of at least 30 kg/m^2 ^who were randomly assigned to receive tirzepatide (up to 15 mg subcutaneously once per week) or placebo for at least 52 weeks.	Primary outcome: additional prespecified clinical endpoints, including sensitivity analyses of the composite of adjudicated CV death or worsening HF. Secondary outcomes: KCCQ-CSS change thresholds (≥20, ≥15, ≥10, and ≥5 points at week 52); 6-MWD change thresholds (≥10, ≥20, and ≥30 m at week 52); change in EQ-5D-5L at 24 and 52 weeks; change in PGIS at 24 and 52 weeks; change in NYHA functional class at 24 and 52 weeks; change in HF medication usage at 52 weeks	Primary outcomes: tirzepatide showed consistent benefit across all sensitivity analyses of composite events endpoint, driven by fewer worsening HF events requiring hospitalization or urgent care IV therapy (HR: 0.41; p<0.05); HRs across analyses ranged from 0.41 to 0.67, favoring tirzepatide. Secondary outcomes: tirzepatide significantly increased the proportion of patients reaching each KCCQ-CSS threshold versus placebo (p<0.01); tirzepatide significantly increased the proportion of patients reaching each 6-MWD threshold versus placebo (p<0.001); tirzepatide significantly increased EQ-5D-5L health state index versus placebo at 24 and 52 weeks (p<0.001); at 24 and 52 weeks, tirzepatide participants were more likely to improve and less likely to worsen in PGIS (p<0.01); at 24 and 52 weeks, tirzepatide participants were more likely to improve and less likely to worsen in NYHA functional class (p<0.01); at 52 weeks, HF medication intensification was less common, and dose reductions were more frequent in the tirzepatide group than in the placebo group (p<0.05). The effect was most pronounced for diuretics, with ~50% fewer dose increases and 50% more dose decreases compared with placebo (p<0.001).
Pratley et al., 2024 [13]	FLOW (HF outcomes analysis)	Prespecified RCT-derived analysis	To investigate prespecified HF outcomes related to semaglutide treatment in patients with T2DM and CKD	This prespecified analysis used the randomized population from the FLOW trial, which enrolled 3,533 patients with T2DM and CKD who were randomly assigned to receive semaglutide or placebo.	Primary outcome: composite of adjudicated CV death or worsening HF, analyzed as time-to-first event. Secondary outcomes: analysis of time-to-first HF event alone excluding CV death; analysis of time-to-first HF hospitalization or urgent IV therapy visit	Primary outcome: semaglutide significantly reduced the risk of CV death or worsening HF versus placebo (HR: 0.73; p<0.001). Secondary outcomes: semaglutide significantly reduced the risk of a first HF event alone versus placebo (p<0.05); semaglutide significantly reduced the risk of first HF hospitalization or urgent visit requiring IV therapy versus placebo (p<0.05).

Patient-Reported Heart Failure Symptoms and Health Status

Patient-reported heart failure symptoms and quality of life were primarily evaluated using KCCQ-CSS. In both STEP-HFpEF and STEP-HFpEF DM trials, semaglutide led to a significantly greater improvement in KCCQ-CSS from baseline to 52 weeks than placebo [9,10].

In the SUMMIT trial, tirzepatide led to a significantly greater improvement in KCCQ-CSS than placebo at 52 weeks [11]. Zile et al. further demonstrated that tirzepatide increased the proportion of patients reaching each KCCQ-CSS threshold (≥5-, ≥10-, ≥15-, and ≥20-point increases) compared with placebo at 52 weeks [12].

Functional Capacity and Exercise Tolerance

Functional capacity and exercise tolerance were assessed using 6-MWD across multiple studies. In the STEP-HFpEF trial, the semaglutide group led to significantly greater improvement in 6-MWD than the placebo group at 52 weeks [9]. In the STEP-HFpEF DM trial, semaglutide similarly led to a significantly greater improvement in 6-MWD than placebo at 52 weeks [10].

In the SUMMIT trial, tirzepatide resulted in a significantly greater improvement in 6-MWD at 52 weeks than placebo [11]. The SUMMIT clinical trajectory analysis further revealed that tirzepatide increased the proportion of patients reaching each 6-MWD threshold (≥10-, ≥20-, and ≥30-m increases) compared with placebo at 52 weeks [12].

Clinical Heart Failure Events and Composite Outcomes

Clinical heart failure events and composite outcomes were assessed in all five studies. In the SUMMIT trial, tirzepatide resulted in a significantly lower incidence of the composite endpoint of cardiovascular death or worsening heart failure event compared with placebo in a time-to-first-event analysis [11]. Additionally, favorable effects of tirzepatide were consistently demonstrated across all sensitivity analyses of the composite endpoint in the prespecified SUMMIT clinical trajectory analysis. The effect of tirzepatide was driven by an effect on worsening heart failure events requiring hospitalization or the use of intravenous medications in an urgent care setting. The hazard ratio for all sensitivity analyses of the primary composite events endpoint ranged from 0.41 to 0.67, consistently favoring tirzepatide [12].

In the STEP-HFpEF trial, analysis of the hierarchical composite endpoint demonstrated more wins in the semaglutide group than in the placebo group, with a stratified win ratio of 1.72. The wins favored semaglutide over placebo across all key components of the composite [9]. The hierarchical composite endpoint similarly favored semaglutide, with a stratified win ratio of 1.58, with wins favoring semaglutide for most key components of the composite in the STEP-HFpEF DM trial [10].

Pratley et al. found that semaglutide resulted in a significantly lower incidence of the composite endpoint of cardiovascular death or worsening heart failure event compared with placebo in patients with T2DM and CKD. Additional analyses demonstrated that semaglutide significantly reduced the risk of a first heart failure event alone and the risk of first heart failure hospitalization or urgent visit requiring intravenous therapy when compared with placebo [13].

Functional Classification and Global Patient-Reported Outcomes

Functional classification and patient-reported global health status were assessed using NYHA functional class, PGIS, and EQ-5D-5L health state index in the SUMMIT clinical trajectory analysis. Zile et al. found that patients taking tirzepatide were more likely to show improving NYHA functional class and less likely to report worsening NYHA functional class at both 24 and 52 weeks compared to placebo. Similarly, the tirzepatide group was more likely to show improvement in PGIS and less likely to report worsening in PGIS at both 24 and 52 weeks compared to placebo. EQ-5D-5L health state index was also significantly improved in patients taking tirzepatide compared with placebo at both 24 and 52 weeks [12].

Changes in Background Heart Failure Therapy

Changes in background heart failure medication use were additionally evaluated in the SUMMIT clinical trajectory analysis. At 52 weeks, intensification of heart failure medications occurred less frequently in the tirzepatide group, whereas the use of these medications was reduced more frequently in the tirzepatide group than in the placebo group. Notable differences in diuretic therapy were observed, including approximately 50% fewer diuretic dose increases and 50% more frequent diuretic dose decreases in the tirzepatide group compared with the placebo group [12].

Discussion

Taken together, the included RCT-based studies suggest that GLP-1-based therapies may be most relevant for cardiometabolic heart failure populations, especially HFpEF with obesity. The consistency of symptom and functional improvements across the HFpEF obesity studies supports a patient-centered benefit in this population, while FLOW suggests that heart failure event reduction may also be relevant in patients with T2DM and CKD [9-13].

A consistent theme across the included studies is that improvements were observed in patient-centered outcomes, including symptoms, functional capacity, and quality of life. This is clinically important because patients with HFpEF often continue to have substantial symptom burden despite standard therapies. The event-based findings from SUMMIT and FLOW also suggest that these benefits may extend beyond symptom improvement, although the populations studied were different and should not be interpreted as interchangeable [11-13].

Comparison With Existing Reviews and Meta-Analyses

Many prior systematic reviews and meta-analyses about GLP-1s have examined atherosclerotic cardiovascular outcomes with heart failure as a secondary or exploratory outcome. Other studies have found neutral or minimal effects of these drugs on heart failure outcomes, indicating a level of uncertainty about the potential benefits of these therapies [18]. More recent reviews of heart failure trials and prespecified analyses have observed positive outcomes, such as improved functional capacity, fewer symptoms and heart failure events, and higher quality of life, in populations with HFpEF and comorbidities. The data from these studies and this scoping review suggest that patients with obesity-related HFpEF may be particularly responsive to GLP-1-based therapies that target cardiometabolic pathways [4,19].

This scoping review contributes additional detail to existing analyses by including broader clinical outcomes and longitudinal measures. The STEP-HFpEF and STEP-HFpEF DM trials observed benefits in composite clinical outcomes, symptoms, and functional capacity, highlighting patient-centered outcomes that may not be fully recognized in meta-analyses that often focus on hospitalization [9,10]. The SUMMIT analysis also showed improvements across various domains throughout time rather than isolated endpoints [11,12]. This review provides a structured and multidimensional synthesis of patient-centered composite heart failure outcomes.

One comprehensive meta-analysis has suggested that GLP-1-based therapies reduce visceral and epicardial fats, leading to improved hemodynamics and heart failure outcomes [20]. The benefits observed in these studies are probably multifactorial. In patients with HFpEF and obesity, weight loss likely contributes to improved symptoms and function, but other effects, such as improved glycemic control, lower inflammation, and reduced cardiac stress related to adiposity, may also play a role. The included studies were not designed to separate the effects of weight loss from other cardiometabolic effects, so it remains unclear how much each mechanism contributes. 

Gaps in the Literature and Directions for Future Research

Although recent studies show encouraging heart failure-specific outcomes, important gaps remain. This review found the strongest evidence in patients with HFpEF and obesity, but it is still unclear whether these findings apply to patients without obesity or those with more advanced heart failure. The included studies also involved different patient populations, which limits how broadly the findings can be applied. STEP-HFpEF, STEP-HFpEF DM, and SUMMIT provide the most direct evidence for patients with HFpEF and obesity, with or without T2DM. FLOW, however, studied patients with T2DM and CKD and reported prespecified heart failure outcomes rather than focusing on a dedicated HFpEF population. Because of these differences, the findings are most applicable to obesity-related HFpEF and other cardiometabolic heart failure populations. Whether similar benefits would be observed in patients with HFrEF remains uncertain. Ongoing studies, including NCT06423599, are looking at semaglutide-based weight loss in patients with obesity and HFrEF, which may help clarify whether these benefits also apply to patients with reduced ejection fraction. Since the existing studies include different heart failure and cardiometabolic populations, treatment effects may vary across patient subgroups [18]. Future trials should help clarify which patients are most likely to benefit from specific GLP-1-based therapies.

Studies may benefit from including extended follow-ups to assess whether improvement in symptoms, functions, adverse events, and quality of life translates into the long term. Many existing trials have limited follow-up periods; however, the use of GLP-1-based therapies for heart failure is relatively new.

There is still a need for a more specific and standardized definition of a heart failure event. In the SUMMIT and FLOW trials, these events were defined as acute clinical deterioration leading to hospitalization or urgent medical visit, requiring treatment [11-13]. Across all studies, this definition may be interpreted slightly differently, influencing the incidence of heart failure events reported.

This scoping review is deliberately descriptive. In the future, when a greater number of heart failure-focused trials of GLP-1-based therapies are conducted, more quantitative syntheses may be useful for certain populations, especially HFpEF with obesity.

Limitations of This Scoping Review

Certain limitations of this review exist and should be acknowledged. This scoping review is qualitative in nature and did not include a formal meta-analysis. This is mainly because five RCT-based studies met inclusion criteria, and the included studies differed in patient populations, interventions, and reported heart failure outcomes. The FLOW trial enrolled a much larger population than the dedicated HFpEF studies, so the narrative synthesis should not be interpreted as giving equal weight to each included study [13]. Two of the included studies are prespecified analyses, so readers must acknowledge that not every included study is a separate RCT [12,13]. The included studies analyzed various drugs, populations, and outcomes, making direct comparisons across trials difficult. Although this scoping review focuses on heart failure outcomes, some observed benefits may be a result of broader cardiometabolic effects, so direct causality cannot be definitively concluded.

Implications for Clinical Practice

Overall, current data suggest that GLP-1-based therapies, especially semaglutide and tirzepatide, may provide meaningful benefits to reduce symptom burden and heart failure events and improve functional capacity and quality of life for patients with HFpEF and obesity. Semaglutide additionally may reduce adverse heart failure events in patients with T2DM and CKD [13]. Notably, the SUMMIT and FLOW trials defined heart failure events using unplanned hospitalizations or urgent care visits requiring therapy escalation, making these findings applicable to the real world [11-13].

In current clinical practice, GLP-1-based therapies play an increasingly important role in managing patients with HFpEF and obesity, a population with high symptom burden and limited treatment options. Findings suggest that tirzepatide may also reduce the need for heart failure medication and diuretic escalation, potentially improving clinical stability [12]. As additional evidence emerges soon, guidelines may clarify the role of GLP-1-based therapies in the treatment of heart failure. In the meantime, the available evidence supports a cautious approach to these therapies alongside already established heart failure treatments.

## Conclusions

Overall, the current evidence suggests that GLP-1-based therapies may be helpful for certain patients with heart failure, especially those with HFpEF and obesity. In the studies included in this review, semaglutide and tirzepatide were linked with improvements in symptoms, walking distance, quality of life, and some heart failure-related outcomes. These findings are encouraging, especially because many patients with HFpEF continue to have a high symptom burden despite available treatments.

However, these findings should still be viewed carefully. The evidence is still developing, and this review included only a small number of contemporary trials and prespecified analyses. Future studies should help clarify which patients benefit the most, whether these improvements last over time, and how GLP-1-based therapies should be used alongside established heart failure treatments.

## References

[1] Gevaert AB, Boen JR, Segers VF, Van Craenenbroeck EM (2019). Heart failure with preserved ejection fraction: a review of cardiac and noncardiac pathophysiology. Front Physiol.

[2] Mentz RJ, Kelly JP, von Lueder TG (2014). Noncardiac comorbidities in heart failure with reduced versus preserved ejection fraction. J Am Coll Cardiol.

[3] Shah KS, Xu H, Matsouaka RA (2017). Heart failure with preserved, borderline, and reduced ejection fraction: 5-year outcomes. J Am Coll Cardiol.

[4] Waqas SA, Sohail MU, Saad M (2025). Efficacy of GLP-1 receptor agonists in patients with heart failure and mildly reduced or preserved ejection fraction: a systematic review and meta-analysis. J Card Fail.

[5] American Diabetes Association (2022). Standards of medical care in diabetes-2022 abridged for primary care providers. Clin Diabetes.

[6] Huixing L, Di F, Daoquan P (2023). Effect of glucagon-like peptide-1 receptor agonists on prognosis of heart failure and cardiac function: a systematic review and meta-analysis of randomized controlled trials. Clin Ther.

[7] Llongueras-Espí P, García-Romero E, Comín-Colet J, González-Costello J (2025). Role of glucagon-like peptide-1 receptor agonists (GLP-1RAs) in patients with chronic heart failure. Biomolecules.

[8] Tricco AC, Lillie E, Zarin W (2018). PRISMA extension for scoping reviews (PRISMA-ScR): checklist and explanation. Ann Intern Med.

[9] Kosiborod MN, Abildstrøm SZ, Borlaug BA (2023). Semaglutide in patients with heart failure with preserved ejection fraction and obesity. N Engl J Med.

[10] Kosiborod MN, Petrie MC, Borlaug BA (2024). Semaglutide in patients with obesity-related heart failure and type 2 diabetes. N Engl J Med.

[11] Packer M, Zile MR, Kramer CM (2025). Tirzepatide for heart failure with preserved ejection fraction and obesity. N Engl J Med.

[12] Zile MR, Borlaug BA, Kramer CM (2025). Effects of tirzepatide on the clinical trajectory of patients with heart failure, preserved ejection fraction, and obesity. Circulation.

[13] Pratley RE, Tuttle KR, Rossing P (2024). Effects of semaglutide on heart failure outcomes in diabetes and chronic kidney disease in the FLOW trial. J Am Coll Cardiol.

[14] (2002). ATS statement: guidelines for the six-minute walk test. Am J Respir Crit Care Med.

[15] Herdman M, Gudex C, Lloyd A (2011). Development and preliminary testing of the new five-level version of EQ-5D (EQ-5D-5L). Qual Life Res.

[16] Rhatigan K, Hirons B, Kesavan H (2023). Patient Global Impression of Severity scale in chronic cough: validation and formulation of symptom severity categories. J Allergy Clin Immunol Pract.

[17] Russell SD, Saval MA, Robbins JL (2009). New York Heart Association functional class predicts exercise parameters in the current era. Am Heart J.

[18] Merza N, Akram M, Mengal A (2023). The safety and efficacy of GLP-1 receptor agonists in heart failure patients: a systematic review and meta-analysis. Curr Probl Cardiol.

[19] Beshr MS, Shembesh RH, Kara AO, Salama AH, Arhaym E, Abuajamieh M, Elhadi M (2025). Efficacy of semaglutide and other GLP-1 agonists in patients with heart failure with preserved ejection fraction and obesity: a systemic review and meta-analysis. Cardiol Rev.

[20] Ahmed M, Burhan M, Shafiq A (2025). GLP-1 and dual GLP-1/GIP receptor agonists in heart failure with mildly reduced or preserved ejection fraction: a systematic review and meta-analysis. Clin Cardiol.

